# Downregulated Plasma‐Derived Extracellular Vesicle MicroRNAs as Novel Biomarkers for Enhanced Tuberculosis Diagnosis

**DOI:** 10.1155/jimr/9206625

**Published:** 2026-08-27

**Authors:** Yuheng Liu, Lei Zhang, Yi Yang, Junwei Wu, Liyuan Wu, Yilin Yang, Haiyi Deng, Wenxiang Jin, Shuwen Wu, Xiaolei Guo, Wenqiang Dang, Jin Su, Junbin Wu, Feifei Liu, Xiaofeng Zou, Yingxin He, Jie Wei, Weihong Wen, Xiaoyan Deng, Yao Liao, Lifu Wang

**Affiliations:** ^1^ The Sixth School of Clinical Medicine, The Affiliated Qingyuan Hospital (Qingyuan People’s Hospital), Guangzhou Medical University, Qingyuan 511518, China, gzhmc.edu.cn; ^2^ KingMed School of Laboratory Medicine, Guangzhou Medical University, Guangzhou 511436, China, gzhmc.edu.cn; ^3^ Engineering Technology Research Center of Intelligent Diagnosis for Infectious Diseases in Guangdong Province, Guangzhou 511436, China; ^4^ Guangzhou Key Laboratory for Clinical Rapid Diagnosis and Early Warning of Infectious Diseases, Guangzhou 511436, China; ^5^ Guangzhou Kingmed Center for Clinical Laboratory, Guangzhou 510320, China; ^6^ The First Affiliated Hospital of Guangzhou Medical University, National Center for Respiratory Medicine, National Clinical Research Center for Respiratory Disease, State Key Laboratory of Respiratory Disease, Guangzhou Institute of Respiratory Health, Guangzhou 510163, China, gzhmc.edu.cn; ^7^ The Department of Clinical Laboratory, The Second Affiliated Hospital of Guangzhou Medical University, Guangzhou 510120, China, gzhmc.edu.cn

**Keywords:** biomarker, diagnosis, extracellular vesicles, microRNA, tuberculosis

## Abstract

**Background:**

Tuberculosis (TB) remains a leading global cause of infectious disease mortality, while current diagnostic methods face substantial limitations. Extracellular vesicle (EV)–encapsulated microRNAs (miRNAs) demonstrate significant potential as diagnostic biomarkers. This study aimed to evaluate their diagnostic utility for TB by identifying expression disparities between TB patients and healthy controls (HCs) and exploring EV miRNA diagnostic performance.

**Methods:**

HCs were healthy individuals excluded from pulmonary infectious diseases, and TB patients were clinically and laboratory‐confirmed cases without other concurrent pulmonary infections. Small RNA sequencing analysis was performed on plasma EVs from a discovery cohort (*n* = 40) to identify differentially expressed (DE) miRNAs. Findings were validated via quantitative real‐time PCR (qRT‐PCR) in an independent cohort (*n* = 24). Subsequently, an EV miRNA combinatorial diagnostic model was developed using random forest classifiers in a modeling cohort (*n* = 58), incorporating feature selection based on importance scores and correlation coefficients. Hyperparameter optimization and model stability verification were performed via grid search with five‐fold cross‐validation. Diagnostic performance was evaluated through receiver operating characteristic (ROC) analysis. Bioinformatics analysis included target gene prediction using miRNA databases, followed by Gene Ontology (GO) and Kyoto Encyclopedia of Genes and Genomes (KEGG) pathway enrichment analyses.

**Results:**

Sequencing revealed 67 significantly DE EV miRNAs, with 92.5% demonstrating marked downregulation in TB patients. This pattern was confirmed by qRT‐PCR validation. Eleven validated miRNAs were used for model construction. Feature selection identified three optimal miRNAs (miR‐19b‐3p, miR‐181b‐5p, and miR‐98‐5p), generating seven combinatorial models. Individual miRNAs exhibited area under the curves (AUCs) > 0.85, while combinations achieved AUCs > 0.89. Notably, the combinatorial signature of miR‐19b‐3p and miR‐181b‐5p demonstrated outstanding diagnostic discrimination (AUC = 1.000; sensitivity and specificity both 100%), with a five‐fold cross‐validated score of 0.964 indicating robust model stability. Bioinformatics analysis revealed that the target genes of these two miRNAs were enriched in immune response pathways, including the Notch signaling pathway and the cyclic adenosine monophosphate (cAMP) signaling pathway.

**Conclusion:**

This EV miRNA signature demonstrates remarkable diagnostic accuracy for TB, offering a clinically actionable solution to diagnostic challenges.

## 1. Introduction

Tuberculosis (TB) has been a leading cause of mortality from infectious diseases globally [1]. A global total of 8.2 million people were reported as newly diagnosed with TB in 2023, surpassing the case numbers recorded from 2020 to 2022 [2]. This upward trend in TB cases worldwide may be related to challenges in achieving early diagnosis and treatment. Identifying active TB (ATB) cases is crucial, yet current diagnostic tests present significant challenges. Routine methods like culturing *Mycobacterium tuberculosis* (*Mtb*) and acid‐fast bacilli smear microscopy are limited by demanding conditions and low detection rates [3, 4]. In recent years, molecular detection methods like Xpert MTB/RIF and Xpert MTB/RIF Ultra, which detect *Mtb* nucleic acids, and interferon‐gamma release assay (IGRA) serological tests, which detect the release of gamma‐interferon by T lymphocytes, have also been widely used in TB diagnosis [5, 6]. However, these methods have their limitations: Xpert MTB/RIF and Xpert MTB/RIF Ultra demonstrate high specificity and sensitivity in smear‐positive cases, yet their diagnostic efficacy is reduced in smear‐negative patients, people living with HIV (PLWH), children, and those with extrapulmonary TB (EPTB) [7]. While IGRA can accurately identify individuals with TB infection, it cannot distinguish between ATB and latent TB infection (LTBI) [8]. The limitations of the current tests highlight an urgent need for new diagnostic methods.

The World Health Organization (WHO) has advocated for TB diagnosis based on biomarkers from nonsputum samples since 2014 [9]. Studies have explored various diagnostic methods, including the detection of *Mtb*‐derived molecules in exhaled breath condensate (EBC), which is rapid but has yet to be clinically validated [10]. Furthermore, the detection of biomarkers in blood is a primary method used in nonsputum specimen diagnostics [11, 12]. Chang et al. [13] identified six plasma cell‐free RNA (cfRNA) biomarkers associated with the host response to TB using RNA sequencing and machine learning and demonstrated their diagnostic potential. The diagnostic accuracy of these six cfRNA gene signatures varied across different datasets, ranging from 88.3% to 91.8% [13]; however, the study’s reliance on specialized equipment such as sequencing platforms limits its applicability in clinical practice.

Extracellular vesicles (EVs) have emerged as novel biomarker carriers with significant research value in disease diagnosis in recent years. These nanoscale vesicles, actively secreted by living cells, carry various functional molecules including RNA and proteins, providing a unique tool for disease monitoring [14–16]. Particularly, microRNAs (miRNAs) are promising candidates for disease diagnosis due to their unique intercellular communication functions [17]. Recently, many studies have focused on the diagnostic role of EV miRNAs, with significant progress in respiratory diseases [18–20]. The EV miRNA regression model developed by Rodrigo‐Muñoz et al. [21] achieved precise classification of asthma patients at different disease stages. Meanwhile, Kim et al. [22] identified three serum EV–specific miRNAs that have proven clinical. Notably, the application of EVs in infectious disease diagnosis remains exploratory, particularly in TB research. It has been reported that macrophages infected with *Mtb* release EVs with differentially expressed (DE) miRNA profiles [23, 24]. Additionally, DE miRNAs have been identified in the serum EVs of patients with LTBI and ATB [25]. These findings demonstrate the differential changes in EV miRNAs associated with TB disease, suggesting their potential diagnostic value.

In this study, we systematically analyzed plasma‐derived EV miRNA expression profiles in TB patients versus healthy controls (HCs) to identify EV miRNA biomarkers with early diagnostic potential. Our small RNA sequencing analysis revealed that 92.5% of 67 significantly DE EV miRNAs exhibited marked downregulation, a pattern subsequently validated in an independent clinical cohort. Notably, through stringent screening criteria, the combinatorial signature of miR‐19b‐3p and miR‐181b‐5p demonstrated exceptional diagnostic performance, achieving an outstanding area under the curve (AUC) of 1.000 (95% CI: 1.000−1.000) with both sensitivity and specificity reaching 100%, indicating optimal diagnostic accuracy.

## 2. Methods

### 2.1. Study Participants

We collected a total of 98 clinical plasma samples from individuals aged above 18 years at the Guangzhou Kingmed Center for Clinical Laboratory from January 2022 to December 2022. The exploratory cohort included 20 HC and 20 TB patients. In the differential expression miRNA validation stage, the cohort included 12 HC and 12 TB patients. For the development of the diagnostic model, we supplemented the validation cohort with an additional 24 HC and 10 TB patients. This newly formed group is referred to as the model development cohort. HCs were selected based on the absence of a TB history, active infections, respiratory diseases, and cancer and were confirmed as healthy during routine medical examinations. In contrast, the TB group included patients who met the clinical diagnostic criteria for TB with positive results from any of the following laboratory tests: acid‐fast staining smear microscopy, *Mtb* culture, or Xpert MTB/RIF nucleic acid amplification test. All cases in this group were microbiologically confirmed and presented with characteristic clinical features of TB and were free of other concurrent respiratory diseases. Ethical approval for this study was granted by the ethics committee of Guangzhou Kingmed Center for Clinical Laboratory (approval number 2022109). The requirement for consent was waived by the ethics committee.

### 2.2. EV Purification

EVs were isolated using differential centrifugation. Initially, human plasma samples were subjected to low‐speed centrifugation (700 × g, 20 min, 4°C) in 15 mL polypropylene tubes utilizing a swinging bucket rotor in a refrigerated centrifuge (Eppendorf 5804R, model A‐4‐44, Germany). The supernatants from this step were subsequently centrifuged at 3500 × g for 20 min at 4°C. Following this, the supernatants were transferred to a 1.5 mL polypropylene tube (Eppendorf, Germany) and centrifuged at 10,000 × g for 40 min at 4°C using a fixed‐angle rotor (model #3331, Thermo Electron Corporation, USA). The final centrifugation step involved ultracentrifugation of the supernatants at 120,000 × g for 120 min at 4°C (Optima L‐100xp, Beckman Coulter, USA) in Quick‐Seal tubes. The resulting EV pellet was then resuspended in PBS and stored at −80°C until further use.

### 2.3. EV Identification

Negative‐staining transmission electron microscopy (TEM) was employed to analyze the EVs. Briefly, EVs were placed on a copper grid and negatively stained with a 3% (w/v) aqueous solution of phosphotungstic acid for 1 min. The grid was then examined using a FEI Tecnai G2 Sprit Twin TEM (FEI, USA).

The EV particles were analyzed using nanoparticle tracking analysis (NTA). The NanoSight NS300 instrument (Malvern Instruments, United Kingdom), equipped with a sCMOS camera, a 488 nm laser, and NTA 3.3 Dev Build 3.3.30 software, was utilized for this analysis, which comprised a total of 749 frames.

Additionally, the EV markers were verified by Western blotting. The EVs were lysed using RIPA buffer with added phosphatase and protease inhibitors (Beyotime, China). Lysates were separated on 15% SDS‐PAGE gels, and the separated proteins were transferred to PVDF membranes. Following the transfer, the membranes were blocked with 5% skim milk. The membranes were then incubated with primary antibodies specific to CD81, Alix, TSG101, and Calnexin (both 1:1000; Abcam, UK) overnight at 4°C. The membranes were then incubated with HRP‐conjugated anti‐IgG secondary antibodies for 2 h at room temperature. Protein bands were detected using an ECL system (BioRad, USA).

### 2.4. RNA Extraction and Reverse Transcription

The total RNA from plasma, EVs, and EV‐depleted plasma (EVs‐free) was extracted using the HiPure Liquid RNA Mini Kit and HiPure Exosome RNA Kits (Magnetec, China), following the manufacturer’s protocol. The concentration of the extracted RNA was determined using a NanoDrop ND‐2000 spectrophotometer (Thermo Scientific, USA). Subsequently, the extracted RNA was reverse‐transcribed into complementary DNA (cDNA) using the 1st Strand cDNA Synthesis Kit (AG, China), in accordance with the provided protocol.

### 2.5. Library Preparation and Sequencing

From the exploratory cohort, we collected 100 μL of plasma EVs from each of the 20 HC and each of the 20 TB patients. The samples were pooled within each group to create two distinct samples: one from the HC and one from the TB, for small RNA sequencing. Total RNA was subsequently extracted from EV fractions using standardized protocols and served as the input material for the small RNA library. RNA purification, reverse transcription, library construction, and sequencing were performed at Shanghai Majorbio Bio‐pharm Biotechnology Co., Ltd. (Shanghai, China) according to the manufacturer’s instructions (Illumina, San Diego, CA). One microgram of total RNA per sample was used as the input material for the small RNA library. Sequencing libraries were generated using the QIAseq miRNA Library Kit (Qiagen), following the manufacturer’s recommendations. The activated 5′ and 3′ adaptors were ligated to the total RNA. Then, the adaptor‐ligated RNA was transcribed into first‐strand cDNA by using reverse transcriptase and a random primer. A PCR reaction was performed using complementary primers for 11–12 cycles, and fragments of appropriate size were isolated with 6% Novex TBE PAGE gels. After quantification with Qubit 4.0, the single‐end RNA‐seq sequencing library was sequenced with the Illumina NovaSeq X Plus sequencer.

### 2.6. Preprocessing and Alignment of Sequencing Data

The raw FASTQ files underwent quality trimming using fastp with default settings to remove adapter sequences [26], reads harboring ambiguous bases (poly‐N), and low‐quality bases (Sanger base quality of <20). Posttrimming, reads were deduplicated and size‐selected to retain only unique sequences within the 18–32 nt range, a size window characteristic of mature miRNAs. The filtered reads were then mapped to the human reference genome (GRCh38/hg38) using Bowtie [27], allowing for up to two mismatches per alignment to accommodate posttranscriptional RNA modifications.

### 2.7. miRNA Annotation and Differential Expression Profiling

Following genome alignment, reads were categorized through a hierarchical annotation pipeline. First, sequences matching known human miRNAs were retrieved from the miRBase 22.0 database (http://www.mirbase.org/). Remaining unmapped reads were subsequently screened against the Rfam and Repbase collections to filter out rRNA, tRNA, snRNA, snoRNA, and repetitive elements. Tags that escaped all prior annotations were subjected to de novo miRNA prediction via mirdeep2 [28], which evaluates both genomic context and RNA secondary structure folding energy to distinguish genuine miRNA precursors from background noise. The abundance of each annotated miRNA was normalized to transcripts per million (TPM) to account for library depth variation.

For differential expression assessment between HC and TB groups, the DEGseq algorithm was employed with the following thresholds set at |log_2_(fold change)| ≥ 1 and adjusted *p*‐value < 0.05 [29].

### 2.8. Target Gene Prediction and Functional Enrichment

The screening strategy for identifying target genes is outlined as follows: Both the software and the databases contribute to the prediction, with the miRanda (v3.3a) software providing a score. A higher score indicates greater confidence in the predicted target genes. We selected target genes that received the miRanda score > 165 and were confirmed by TargetScan (v7.0), starBase, and miRTarBase. Functional annotation of predicted targets was subsequently conducted through Gene Ontology (GO) and Kyoto Encyclopedia of Genes and Genomes (KEGG) pathway enrichment analyses.

### 2.9. Quantitative Real‐Time PCR (qRT‐PCR) Assay

EV miRNA expression was measured using the Premix Pro Taq HS qPCR Kit (AG, China) and miRNA‐specific primers. Each 25 μL reaction mixture included 2 μL of template cDNA, 1 μL of miRNA‐specific primer, 1 μL of miRNA qPCR 3′ primer, 12.5 μL of 2 × SYBR Green Pro Taq HS Premix (AG, China), and 8.5 μL of RNase‐free water. Using the universal housekeeping gene RNU6B as the endogenous reference, the primers are listed in Table S1. The qRT‐PCR reactions were conducted with the following cycling parameters: initial step at 95°C for 10 s, following 95°C for 5 s; 60°C for 20 s, with a total of 40 amplification cycles; and a melting curve analysis consisting of 95°C for 60 s, 55°C for 30 s, and 95°C for 30 s. Relative expression levels were calculated using the 2^−ΔΔCt^ method.

### 2.10. Development of Diagnostic Models

In the development phase of the diagnostic models, we employed a random forest classifier implemented via the Python scikit‐learn script to construct an optimal miRNA–based diagnostic model for discriminating between HC and TB patients. Using DE EVs miRNA qRT‐PCR data from the model development cohort (*n* = 58), the dataset was randomly split into a training set (60%) and a testing set (40%) for model training and independent validation, respectively. Hyperparameter optimization was performed through grid search (GridSearchCV) with five‐fold cross‐validation, identifying optimal parameters: maximum depth unrestricted (max_depth = None), maximum features as square root (max_features = ‘sqrt’), minimum samples per leaf node 1 (min_samples_leaf = 1), minimum samples for node splitting 2 (min_samples_split = 2), and 100 decision trees (n_estimators = 100). The model built with these parameters was verified for stability via five‐fold cross‐validation. To mitigate overfitting risks and simplify the model through feature reduction, we performed feature selection based on importance scores. Features exceeding the importance threshold of 0.1 with low interfeature correlation were strategically selected as candidate miRNAs. Through systematic random combination of these filtered miRNAs and reconstruction of random forest classifiers, we evaluated the diagnostic performance using receiver operating characteristic (ROC) analysis. This process ultimately identified the miRNA combination demonstrating optimal diagnostic efficacy.

### 2.11. Statistical Analysis

Results are presented as mean ± SD. Statistical differences between two groups were assessed using Mann–Whitney *U* test or unpaired two‐sample *t*‐tests. *p*‐Values < 0.05 were deemed statistically significant. All statistical analyses were performed using GraphPad Prism version 9.0 (GraphPad Software, USA).

## 3. Results

### 3.1. HC and TB Patients’ Characteristics and Identification of Plasma‐Derived EVs

**Figure 1:**
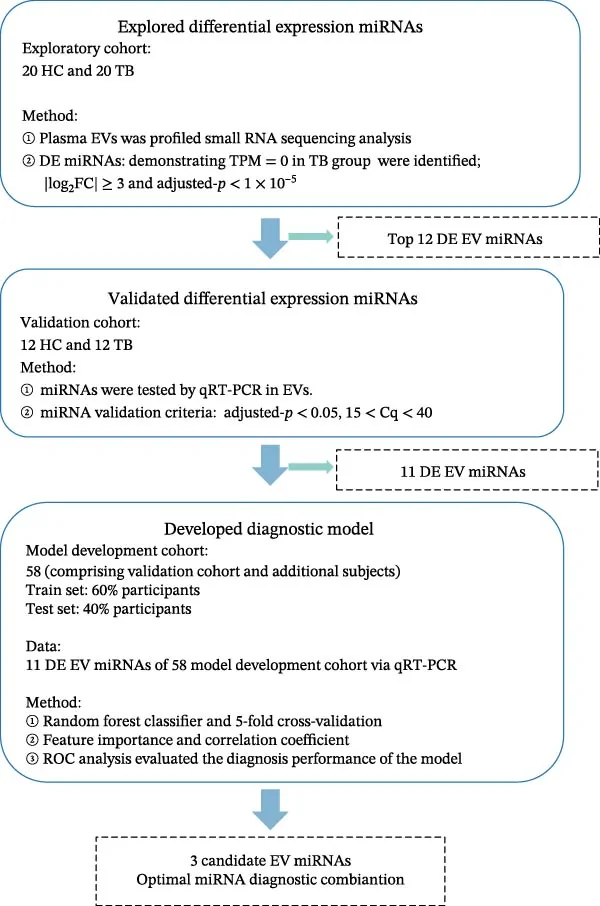
Overview of the strategy for investigating EV miRNAs and developing a diagnostic model for TB. This schematic shows the workflow for investigating EV miRNA profiles and developing a TB diagnostic model, initiating with small RNA sequencing of plasma EVs in the exploratory cohort, which identified 12 DE miRNAs (log_2_FC ≥ 3, adjusted‐*p* < 1*×*10^−5^). Independent qRT‐PCR validation confirmed 11 EV miRNAs meeting technical thresholds (adjusted‐*p* < 0.05, 15 < Cq < 40). During model development, the validated miRNAs were analyzed in the model development cohort using a random forest classifier with five‐fold cross‐validation. Feature selection prioritized miRNAs exhibiting high importance scores (≥0.1) and low interfeature correlation, ultimately identifying the optimal diagnostic combination through ROC analysis. Abbreviations: Cq, cycle of quantification; DE, differentially expressed; EV, extracellular vesicle; HCs, healthy controls; qRT‐PCR, quantitative real‐time polymerase chain reaction; ROC, receiver operating characteristic; TB, tuberculosis.

The flow chart of the study is shown in Figure 1. During the “developed diagnostic model stage,” we enrolled a total of 58 participants, comprising both the validation cohort and additional subjects. The validation cohort included 12 HC (eight males and four females, mean age: 42.75 ± 15.08 years) and 12 TB patients (seven males and five females, mean age: 46.25 ± 14.26 years). The model development cohort consisted of 36 HC (15 males and 21 females, mean age: 41.61 ± 13.63 years) and 22 TB patients (14 males and eight females, mean age: 47.32 ± 15.29 years). Clinical characteristics for the 58 participants included in the development of the diagnostic model are detailed in Table S2.

**Figure 2:**
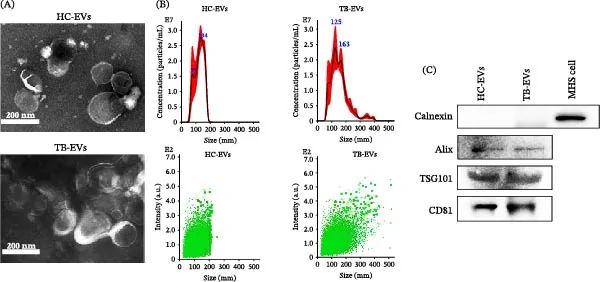
Characterization of plasma‐derived EVs from HC and TB patients. (A) Plasma EVs from HC (HC‐EVs) and TB patients (TB‐EVs) were analyzed by negative‐staining transmission electron microscopy (TEM). (B) Size distribution of EV particles was investigated using nanoparticle tracking analysis (NTA). (C) Western blotting identified the expression of EV biomarkers Calnexin, Alix, TSG101, and CD81 in plasma‐derived EVs, MHS cell served as a positive control for the detection of Calnexin.

EVs were successfully isolated from plasma samples and identified using TEM, NTA, and Western blotting. TEM revealed that the EVs displayed the characteristics of cup‐shaped nanovesicles (Figure 2A). NTA indicated that the size distribution of the majority of vesicles isolated from plasma ranged from 30 to 200 nm (Figure 2B), which aligns with the normal size range for EVs [14]. Furthermore, the expression of EV markers Alix, TSG101, and CD81 was assessed in both HC‐EV and TB‐EV samples by Western blotting (Figure 2C). As an endoplasmic reticulum–resident protein and a widely used negative marker for EV preparations, Calnexin was not detected in the samples, confirming the absence of cellular contamination in the isolated EVs (Figure 2C).

### 3.2. Plasma‐Derived EV‐miRNAs in TB Patients Were Significantly Downregulated

**Figure 3:**
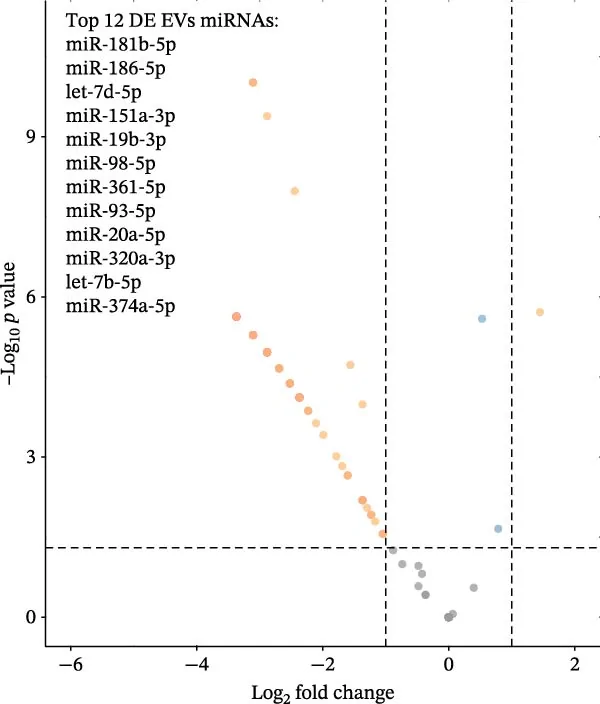
Twelve candidate EV miRNAs were selected from the exploratory cohort. The volcano plot displays 150 miRNAs identified in plasma EVs from the exploratory cohort. Candidate DE miRNAs were selected through stringent criteria: (1) TPM = 0 in the TB group, (2) |log_2_FC| ≥ 3, and adjusted *p*‐value < 1 × 10^−5^. The 12 candidate EV miRNAs meeting these criteria are highlighted, representing the most significant DE for further validation.

To identify EV miRNAs with diagnostic potential for TB, we established an exploratory cohort comprising plasma samples from 20 HC and 20 TB patients for small RNA sequencing analysis. The sequencing revealed 150 distinct miRNAs in plasma EVs across both groups. Differential expression analysis employing statistical thresholds (adjusted *p*‐value < 0.05 with |log2FC| ≥ 1) identified 67 significantly DE miRNAs between HC and TB groups. Notably, 62 miRNAs (92.5%) exhibited marked downregulation in TB‐EVs compared to HC counterparts, as visualized in the volcano plot depicting miRNA expression profiles (Figure 3).

To prioritize candidate DE miRNAs, we implemented a two‐stage filtration protocol: first, miRNAs demonstrating complete absence of expression in the TB group (TPM = 0) were identified; second, these candidates were further refined using enhanced stringency criteria (|log2FC| ≥ 3 and adjusted *p*‐value < 1 × 10^−5^). This hierarchical screening identified 12 top‐ranked EV miRNAs exhibiting the most pronounced DE: miR‐181b‐5p, 186‐5p, let‐7d‐5p, 151a‐3p, 19b‐3p, 98‐5p, 361‐5p, 93‐5p, 20a‐5p, 320a‐3p, let‐7b‐5p, and 374a‐5p (Figure 3).

### 3.3. DE EV miRNAs Were Predominantly Downregulated in TB‐EVs

**Figure 4:**
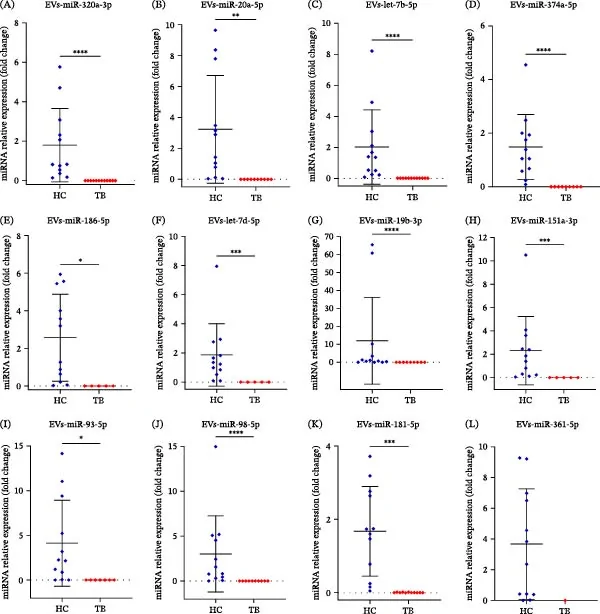
DE EV‐miRNA downregulation in TB‐EVs. qRT‐PCR was performed using plasma‐derived EVs from 12 HC and 12 TB patients. (A–L) Expression levels of 12 DE EV miRNAs in HC‐EVs and TB‐EVs (*n* = 12). The data were analyzed using Mann–Whitney *U* test or unpaired two‐sample *t*‐tests, comparing each experimental condition with the control. RNU6B was used as an endogenous reference. Data are mean ± SDs;  ^∗^ *p* < 0.05;  ^∗∗^ *p* < 0.01;  ^∗∗∗^ *p* < 0.001; and  ^∗∗∗∗^ *p* < 0.0001.

To validate the candidate DE EV miRNAs, we analyzed clinical samples from a validation cohort comprising 12 HC and 12 TB patients. Independent validation was performed using qRT‐PCR to detect target miRNAs in EV samples, with RNU6B as the endogenous reference. The analysis revealed that 11 EV‐associated miRNAs (miR‐181b‐5p, 186‐5p, let‐7d‐5p, 151a‐3p, 19b‐3p, 98‐5p, 93‐5p, 20a‐5p, 320a‐3p, let‐7b‐5p, and 374a‐5p) demonstrated significantly lower expression levels in TB patients compared to HC (Figure 4A–K). miR‐361‐5p was excluded from subsequent analyses due to its low detection rate in the validation cohort (Figure 4L).

To investigate whether the candidate miRNA expression observed between TB and HC was primarily associated with EVs, we performed qRT‐PCR analysis on paired plasma and EVs‐free from individuals in both cohorts. While 11 miRNAs showed reduced expression in TB plasma compared to HC, the magnitude of downregulation was less pronounced than that observed in EVs (Figure S1A, C–E, G–K). Notably, miR‐20a‐5p and let‐7d‐5p exhibited no significant differential expression in plasma (Figure S1B,F). In EVs‐free, six miRNAs (let‐7b‐5p, miR‐186‐5p, 19b‐3p, 181b‐5p, 98‐5p, and 151a‐3p) had no significant group differences (Figure S2C, E, G, H, J, K), while miR‐320a‐3p and miR‐20a‐5p maintained differential (Figure S2A, B). Three miRNAs (miR‐374a‐5p, 93‐5p, and let‐7d‐5p) fell below the detection threshold (Cq > 40) in EVs‐free due to low expression levels (Figure S2D,F,I). These findings demonstrate that the significant downregulation of candidate miRNAs in TB patients predominantly occurs within EVs, suggesting that the observed reductions in plasma miRNA levels are largely due to decreased EV‐associated miRNA content. This EV‐specific expression pattern highlights the diagnostic potential of EV‐encapsulated miRNAs for TB detection. Based on these validation results, we selected the 11 DE EV miRNAs for subsequent investigations.

### 3.4. EV miRNA Combination Diagnostic Model Enhances TB Diagnostic Accuracy

**Figure 5:**
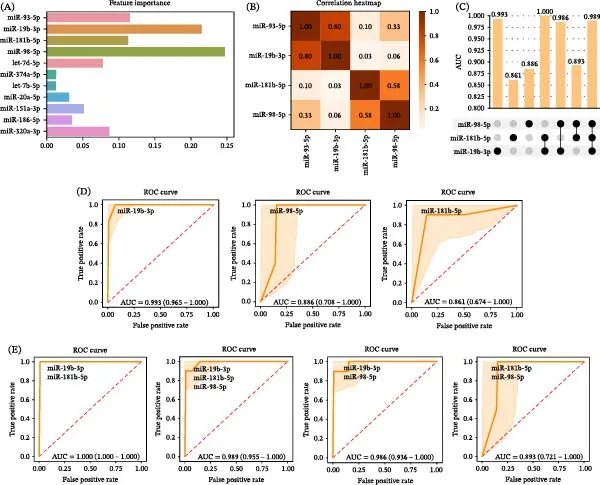
EV miRNA combination diagnostic model enhances TB diagnostic accuracy. (A) Feature importance highlights the significance of the 11 EV miRNAs to the model. (B) The heatmap displays the correlation coefficients among miR‐98‐5p, miR‐181b‐5p, miR‐19b‐3p, and miR‐93‐5p, with darker shades indicating stronger correlations. (C) The area under the receiver operating characteristic (ROC) curve (AUC) for individual EV miRNAs and EV miRNA combinations. ROC curves for classifying plasma‐derived EVs, generated using expression values of (D) miR‐19b‐3p, 98‐5p, and 181b‐5p individually, and for (E) three pairwise combinations (miR‐19b‐3p and 181b‐5p, miR‐19b‐3p and 98‐5p, miR‐181b‐5p and 98‐5p) and one triple combination (miR‐19b‐3p, 181b‐5p, and 98‐5p).

To assess the diagnostic feasibility of EV miRNAs for TB, we established a random forest classifier model incorporating 11 DE EV miRNAs quantified by qRT‐PCR in a model development cohort (*n* = 58). The cohort data were randomly partitioned into training (60%) and testing (40%) sets, revealing four EV miRNAs (miR‐98‐5p, miR‐181b‐5p, miR‐93‐5p, and miR‐19b‐3p) with feature importance > 0.1 (Figure 5A). Correlation analysis demonstrated a strong positive association between miR‐93‐5p and miR‐19b‐3p (*r* = 0.8, *p* < 0.0001; Figure 5B). Given the higher feature importance of miR‐19b‐3p compared to miR‐93‐5p (Figure 5A), we prioritized miR‐19b‐3p for subsequent analyses. This selection process ultimately identified three candidate EV‐miRNAs (miR‐98‐5p, miR‐181b‐5p, and miR‐19b‐3p) as candidate miRNAs for constructing diagnosis combinations.

The ROC curve graphically illustrates the diagnostic test performance by plotting true positive rate (TPR, sensitivity) against false positive rate (FPR, 1–specificity) [30]. We systematically evaluated random combinations of three candidate miRNAs (miR‐98‐5p, miR‐181b‐5p, and miR‐19b‐3p) through random forest classifier construction and ROC analysis with AUC calculation (Figure 5C). Among individual miRNAs, miR‐19b‐3p demonstrated the strongest performance with an AUC of 0.993 (95% CI: 0.965−1.000) (Figure 5D). Four combinatorial configurations were generated from these miRNAs: three pairwise combinations (miR‐19b‐3p and miR‐181b‐5p; miR‐19b‐3p and miR‐98‐5p; miR‐181b‐5p and miR‐98‐5p) and one triple combination (miR‐19b‐3p, miR‐181b‐5p, and miR‐98‐5p). Notably, the pairwise combination of miR‐19b‐3p and miR‐181b‐5p achieved a perfect AUC of 1.000 (95% CI: 1.000−1.000), outperforming individual miRNAs and other combinatorial configurations (Figure 5E). ROC analysis revealed that this optimal pair attained 100% specificity and sensitivity, representing the highest diagnostic accuracy among all evaluated combinations (Table 1). Comparative analysis of the four miRNA combinations based on AUC values, specificity, and sensitivity demonstrated that the miR‐19b‐3p and miR‐181b‐5p pair exhibited superior diagnostic performance for distinguishing HCs from TB patients. These findings suggest that the EV‐miRNA combinatorial signature can significantly enhance diagnostic efficacy, providing exceptional accuracy and robust discriminatory power.

**Table 1:** Sensitivity and specificity of miRNA combinations in diagnosing TB.

miRNA combinations	AUC	Sensitivity	Specificity	Accuracy	Cross‐validated scores
miR‐19b‐3p and miR‐181b‐5p	1.000	1.000	1.000	0.917	0.964
miR‐19b‐3p and miR‐98‐5p	0.986	0.900	1.000	0.958	0.947
miR‐181b‐5p and miR‐98‐5p	0.893	1.000	0.857	0.917	0.930
miR‐19b‐3p and miR‐181b‐5p and miR‐98‐5p	0.989	0.900	1.000	0.917	0.964

### 3.5. EV miRNA Target Gene Prediction

**Figure 6:**
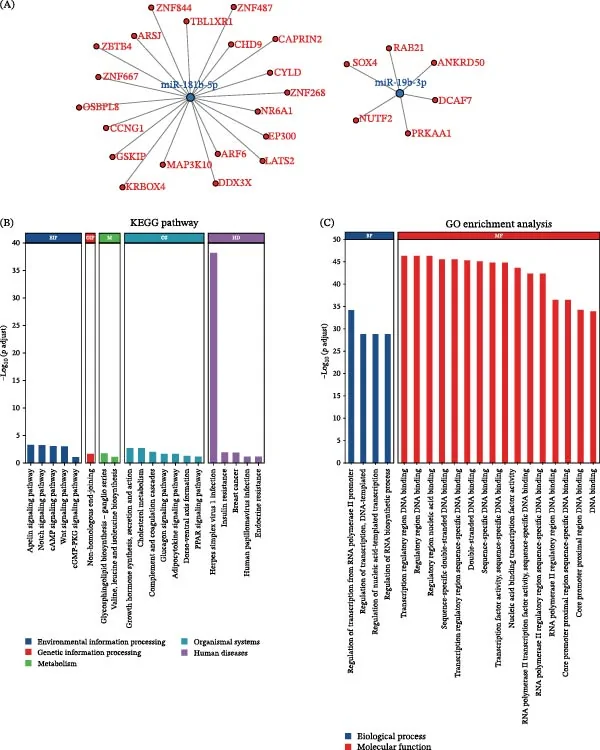
EV miRNAs target gene prediction and enrichment analysis. (A) Interaction networks depicting the interplay between the EV miRNA combination (miR‐19b‐3p and miR‐181b‐5p) and their target genes. (B) Kyoto Encyclopedia of Genes and Genomes (KEGG) pathway analysis for the target genes of the EV miRNA combination. (C) Gene Ontology (GO) enrichment analysis for the functional evaluation of the target genes associated with EV miRNAs.

We predicted the target genes for the optimal EV miRNA combination (miR‐19b‐3p and 181b‐5p). A total of 20 target genes were predicted for miR‐181b‐5p and six for miR‐19b‐3p (Figure 6A). KEGG analysis revealed significant enrichment of the target genes in 153 pathways associated with human diseases, environmental information processing, and metabolism. Notably, the target genes were markedly enriched in pathways related to viral infections, the apelin signaling pathway, the Notch signaling pathway, and the Cyclic adenosine monophosphate (cAMP) signaling pathway (Figure 6B). Further, GO term enrichment indicated that the target genes were involved in 910 biological processes and molecular functions, with significant enrichment in protein binding, binding, biological regulation, and the regulation of biological processes (Figure 6C). Collectively, these findings suggest that the target genes of miR‐181b‐5p and miR‐19b‐3p may play a role in modulating host immune responses following TB infection, although this hypothesis requires further validation.

## 4. Discussion

In this study, we utilized small RNA sequencing to reveal DE miRNAs in EVs from TB patients compared to HC. Our analysis indicates that these DE miRNAs have the potential to serve as diagnostic biomarkers for TB. Specifically, we constructed a diagnostic model incorporating 11 DE miRNAs and found that a particular combination of miRNAs (miR‐181b‐5p and miR‐19b‐3p, AUC = 1, 95% CI: 1.000−1.000) demonstrated superior diagnostic efficacy. Overall, our research not only proposes EV biomarkers with diagnostic potential for TB but also effectively distinguishes between HC and TB patients.

Although research on EV miRNA for TB diagnosis is still scarce, the potential is substantial. Hu et al. [31] reported a diagnostic model that combines exosomal miRNAs with electronic health data to distinguish PTB from tuberculous meningitis (TBM), demonstrating excellent diagnostic performance (AUC 0.99 for PTB; AUC 0.98 for TBM). Similarly, Sun et al. [32] found that miR‐125b‐5p is significantly upregulated in macrophage exosomes from spinal TB, with good diagnostic efficacy (AUC = 0.88). More recently, a study based on droplet digital PCR (ddPCR) identified a three‐EV miRNA panel (hsa‐miR‐451a, hsa‐miR‐1908‐5p, and hsa‐miR‐1268b) that yielded an AUC of 0.970 for discriminating ATB from HCs [33]. Consistent with these published findings, our results further reveal the substantial diagnostic potential of circulating EV miRNAs for TB. In our study, EV miRNAs possess substantial diagnostic potential, with the diagnostic efficacy of a single miRNA reaching as high as 0.993. Furthermore, we have determined that the combined diagnosis using plasma EV miRNAs from TB patients can enhance diagnostic efficacy, with the combination of miR‐19b‐3p and miR‐181b‐5p achieving superior diagnostic performance (AUC = 1, sensitivity and specificity both 100%). The reliable diagnostic performance of EV miRNAs is attributed to their capacity to cross biological barriers and their circulatory stability. The bilayer membrane structure of EVs protects miRNAs from degradation in the bloodstream and allows these molecules to sensitively reflect miRNA expression changes in distant pathological sites.

Beyond miRNA‐based detection approaches, researchers have explored a variety of other nonsputum biomarkers for TB diagnosis. Chang et al. [13] identified a plasma cfRNA signature consisting of six genes, which yielded AUC values of 0.95, 0.92, and 0.95 for discriminating TB‐positive from TB‐negative populations in the test, training, and validation cohorts, respectively. Although cfRNA shows application potential, its detection relies on RNA sequencing platforms, which limits large‐scale deployment in high‐TB‐burden regions with scarce medical resources. In comparison, the EV miRNA signature from our study can be detected via qRT‐PCR, a widely accessible, low‐cost, and relatively straightforward technique that can be directly implemented in clinical laboratories.

In this work, EVs were isolated by ultracentrifugation, a widely adopted and well‐recognized gold‐standard method for EV purification that ensures high sample quality and reliable miRNA expression profiling results [34]. However, ultracentrifugation requires specialized ultracentrifuge equipment, which may not be available in medical institutions in remote areas. For routine clinical translation, alternative clinically feasible EV isolation strategies such as commercial extraction kits can be adopted. These kits do not require dedicated ultracentrifuge instruments, have relatively simple operating workflows, and are more compatible with clinical practice settings.

*Mtb*, the causative agent of TB, has evolved sophisticated strategies to evade host immunity over thousands of years of human infection [35]. As an intracellular pathogen, once engulfed by immune cells such as macrophages, *Mtb* employs proteins like NdkA, CpsA, and PPE2 to disrupt the “LC3‐associated phagocytosis” pathway, a crucial process in autophagy that would otherwise target the bacteria for destruction [36–39]. Moreover, *Mtb* has strategies to evade adaptive immunity: infected dendritic cells (DCs) export *Mtb* antigens via kinesin 2–dependent vesicular transport, diverting them away from major histocompatibility complex class II (MHC‐II) presentation and preventing effective T‐cell activation [40]. The relationship between miRNA and immune regulation is crucial in understanding host responses to bacterial infections [41]. It has been observed that *Mtb* alters the expression of certain host miRNAs, such as miR‐132 and miR‐26a, thereby dampening the immune response [42]. Our study builds on this understanding by revealing a significant downregulation of miRNAs in plasma‐derived EVs from TB patients compared to HC, aligning with findings reported by Singh et al. [23]. We hypothesize that this downregulation may be associated with the immune evasion strategy of *Mtb*. Postinfection, *Mtb* may modulate the release of miRNAs within host EVs, which are then transported via the circulatory system to distant immune cells, influencing their immune responses and achieving potent immune evasion. The alteration of specific host miRNA expression by *Mtb* infection suggests the potential utility of miRNAs as diagnostic markers for TB.

Based on the significant downregulation of TB‐EV miRNA levels, our study ultimately selected a combination of miR‐19b‐3p and miR‐181b‐5p for the diagnosis of TB. Interestingly, these miRNAs have been found to be upregulated in other diseases, such as Newcastle disease virus infection in DF‐1 cells [43], chronic HBV infection [44], and in conditions like acute kidney injury [45], lung adenocarcinoma [46], colorectal cancer [47], and colitis [48]. This contrast in expression patterns between TB and other diseases emphasizes the specificity of miR‐19b‐3p and miR‐181b‐5p as TB markers. Functional enrichment analysis in our study indicated that the target genes of miR‐19b‐3p and miR‐181b‐5p were significantly enriched in pathways including the Notch signaling pathway and cAMP signaling pathway, both of which are closely associated with immune regulation during pathogen infection [49, 50]. Notably, the Notch signaling pathway has been shown to exert critical regulatory effects on macrophage inflammation and phagocytic function during *Mtb* infection [51–53]. Prior studies have confirmed that activation of the Notch1 pathway in *Mtb*‐infected macrophages increases the phosphorylation levels of Akt/mTOR and the nuclear factor‐κB (NF‐κB) p65 subunit, which in turn promotes inflammasome activation and ultimately drives macrophage inflammatory responses [54], supporting an inflammatory regulatory role of Notch1 signaling in the context of *Mtb* infection. Similarly, the cAMP signaling pathway represents a canonical axis that governs macrophage anti‐*Mtb* responses. Upon host infection, the *Mtb* adenylate cyclase Rv0386 converts adenosine triphosphate (ATP) to cAMP, which elevates intracellular cAMP levels in host macrophages and suppresses host immune responses [55]. A separate study demonstrated that the *Mtb* virulence regulator PhoP sustains intracellular cAMP levels by directly inhibiting the transcription of the cAMP‐specific phosphodiesterase Rv0805, thereby enhancing the intracellular survival capacity of *Mtb* in macrophages and uncovering a novel mechanism underlying *Mtb* intracellular persistence following macrophage infection [56]. Taken together, these findings suggest that the target genes of miR‐19b‐3p and miR‐181b‐5p may be involved in the regulation of host immune responses during TB infection, highlighting the unique advantages of these two miRNAs in TB diagnosis and their potential for accurate detection of the disease.

This study has several limitations that should be acknowledged: (1) sample size constraints in exploratory phases necessitate validation in larger cohorts; (2) independent external validation remains pending; (3) prospective clinical implementation studies are required; and (4) expansion of cohorts including young and elderly populations is required to validate the diagnostic efficacy of the model in these age groups.

## 5. Conclusion

**Figure 7:**
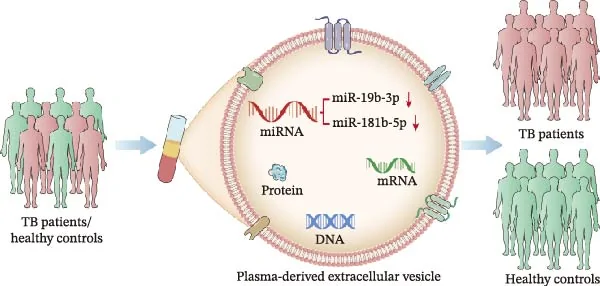
Downregulation of plasma‐derived extracellular vesicle miRNAs as novel biomarkers for enhanced tuberculosis diagnosis. Plasma EV miRNAs were globally downregulated in TB patients relative to HC. A machine learning–based diagnostic model identified the miR‐19b‐3p and miR‐181b‐5p combination as the optimal diagnostic signature, achieving an AUC of 1.000 with 100% sensitivity and specificity.

In summary, we observed significant downregulation of miRNAs in plasma‐derived EVs from TB patients compared to HC. Utilizing machine learning algorithms, we developed a miRNA combination diagnostic model. After a rigorous selection process, we identified the combination of miR‐19b‐3p and miR‐181b‐5p (AUC = 1, sensitivity and specificity, both 100%) as a potential diagnostic miRNA combination for TB (Figure 7).

**Nomenclature**
TB: Tuberculosis
EVs: Extracellular vesicles
miRNAs: MicroRNAs
DE: Differentially expressed
qRT‐PCR: Quantitative real‐time PCR
GO: Gene Ontology
KEGG: Kyoto Encyclopedia of Genes and Genomes
ROC: Receiver operating characteristic
AUC: Area under the curve
ATB: Active TB
*Mtb*: *Mycobacterium tuberculosis*
IGRAs: Interferon‐gamma release assays
PLWH: People living with HIV
EPTB: Extrapulmonary TB
LTBI: Latent TB infection
WHO: World Health Organization
EBC: Exhaled breath condensate
cfRNA: Cell‐free RNA
HCs: Healthy controls
TEM: Transmission electron microscopy
NTA: Nanoparticle tracking analysis
TPM: Transcripts per million
EVs‐free: EV‐depleted plasma
TPR: True positive rate
FPR: False positive rate
TBM: Tuberculous meningitis
cAMP: Cyclic adenosine monophosphate
NF‐κB: Nuclear factor‐κB
ATP: Adenosine triphosphate.

## Author Contributions

Lifu Wang, Yao Liao, Xiaoyan Deng, Yuheng Liu, Lei Zhang, and Weihong Wen conceived and designed the experiments. Yuheng Liu, Yi Yang, Junwei Wu, Liyuan Wu, Yilin Yang, Haiyi Deng, Jin Su, and Yingxin He performed the experiments. Lifu Wang, Lei Zhang, Xiaoyan Deng, Weihong Wen, Wenxiang Jin, Shuwen Wu, Xiaolei Guo, Wenqiang Dang, Junbin Wu, Feifei Liu, and Xiaofeng Zou provided critical samples/materials and reagents/protocols. Yuheng Liu, Yao Liao, and Junwei Wu analyzed the data and performed the statistical analysis and modeling. Yuheng Liu, Lifu Wang, Yao Liao, Lei Zhang, and Yi Yang drafted the initial manuscript.

## Funding

This work was supported by the Science and Technology Plan Project of Guangzhou (Grants 2023A04J0559 and 2025A03J3626), the Department of Education of GuangDong Province (Grant 2023ZDZX2048), the National Natural Science Foundation of China (Grant 81902081), the Natural Science Foundation of Guangdong Province (Grants 2020A1515011573 and 2023A1515220167), Open Project of Guangzhou Medical University, the Guangzhou Key Laboratory for Clinical Rapid Diagnosis and Early Warning of Infectious Diseases (Grant 202102100003), Guangzhou Science and Technology Fund (Grant 2024A03J0791), the Youth Innovation Talent Project of Ordinary University in Guangdong Province (Grant 2022KQNCX061), and Tertiary Education Scientific Research Project of Guangzhou Municipal Education Bureau (Grant 2024312085).

## Disclosure

All authors have critically read and approved the final version of the manuscript. All authors of this study agreed to publish.

## Ethics Statement

Ethical approval for this study was granted by the ethics committee of Guangzhou Kingmed Center for Clinical Laboratory (Approval Number 2022109).

## Conflicts of Interest

The authors declare no conflicts of interest.

## Supporting Information

Additional supporting information can be found online in the Supporting Information section.

## Supporting information


**Supporting Information** Figure S1: 11 DE EVs miRNAs were tested by qRT‐PCR in plasma samples. Figure S2: 11 DE EVs miRNAs were tested by qRT‐PCR in EVs‐free supernatant (EVs‐free) samples. Table S1: Primers used for qRT‐PCR. Table S2: Clinical information of participants.

## Data Availability

The data that support the findings of this study are available from the corresponding author upon reasonable request.
