# Supplementary material for: Downregulated Plasma‐Derived Extracellular Vesicle MicroRNAs as Novel Biomarkers for Enhanced Tuberculosis Diagnosis
Source: J Immunol Res. 2026 Aug 27;2026:9206625. doi: 10.1155/jimr/9206625 (PMC13520784; doi:10.1155/jimr/9206625)
Supplement: Supplementary file 1 [file JIMR-2026-9206625-s001.docx]

**Supplemental material**

**Supplemental Figures AND Figure Legends**

**Figure S1. 11 DE EVs miRNAs were tested by qRT-PCR in plasma samples.** (A-K) Expression levels of 11 DE EVs miRNAs (miR-181b-5p, 186-5p, let-7d-5p, 151a-3p, 19b-3p, 98-5p, 93-5p, 20a-5p, 320a-3p, let-7b-5p, and 374a-5p) in plasma of HC and TB patients (n=12). The data were analyzed using Mann-Whitney U test or unpaired two-sample *t*-tests, comparing each experimental condition with the control. RNU6B was used as an endogenous reference. Data are mean ± SDs; **p* < 0.05; ***p* < 0.01; ****p* < 0.001; *****p* < 0.0001

**Figure S2. 11 DE EVs miRNAs were tested by qRT-PCR in EVs-free supernatant (EVs-free) samples.** (A-K) Expression levels of 11 DE EVs miRNAs in EVs-free of HC and TB patients (n=12). The data were analyzed using Mann-Whitney U test or unpaired two-sample *t*-tests, comparing each experimental condition with the control. RNU6B was used as an endogenous reference. Data are mean ± SDs; **p* < 0.05; ***p* < 0.01; ****p* < 0.001; *****p* < 0.0001

**Table S1. Primers used for qRT-PCR**

| **Gene** | **Forward (5'-3')** | **Reverse (5'-3')** |
| --- | --- | --- |
| miR-93-5p | CAACAAAGTGCTGTTCGTGC | miRNA qPCR 3’ primer |
| miR-181b-5p | ATTCATTGCTGTCGGTGGGT |  |
| miR-98-5p | GGGTGAGGTAGTAAGTTGTAT |  |
| miR-361-5p | TTATCAGAATCTCCAGGGGTA |  |
| miR-151a-3p | CTAGACTGAAGCTCCTTGAG |  |
| miR-19b-3p | GTGCAAATCCATGCAAAACTG |  |
| let-7d-5p | GAGGTAGTAGGTTGCATAGTT |  |
| miR-186-5p | CAAAGAATTCTCCTTTTGGGC |  |
| miR-374a-5p | CCGCCTTATAATACAACCTGA |  |
| let-7b-5p | GAGGTAGTAGGTTGTGTGGT |  |
| miR-320a-3p | AAAGCTGGGTTGAGAGGGC |  |
| miR-20a-5p | AAGTGCTTATAGTGCAGGTAG |  |

**Table S2. Clinical information of participants**

| Variabls | Validation cohort | |  | Model development cohort | |
| --- | --- | --- | --- | --- | --- |
|  | Healthy controls | Tuberculosis |  | Healthy controls | Tuberculosis |
| **Patients, n** | 12 | 12 |  | 36 | 22 |
| **Age** | 42.75±15.08 | 46.25±14.26 |  | 41.61±13.63 | 47.32±15.29 |
| **Gender** |  |  |  |  |  |
| male, n (%) | 8 (66.67%) | 7 (58.33%) |  | 15 (41.67%) | 14 (63.64%) |
| Female, n (%) | 4 (33.33%) | 5 (41.67%) |  | 21 (58.33%) | 8 (36.36%) |

Data represent mean (± SD)
